# Universal credit trajectories among individuals who access secondary mental health services: analysis of linked data

**DOI:** 10.1007/s00127-025-02930-3

**Published:** 2025-05-30

**Authors:** Sharon A. M. Stevelink, Sarah Ledden, Ioannis Bakolis, Ray Leal, Ira Madan, Matthew Hotopf, Nicola T. Fear, Thomas Lorentzen

**Affiliations:** 1 Department of Psychological Medicine, Institute of Psychiatry, Psychology and Neuroscience, King’s College London, London, UK; 2 Department of Psychological Medicine, Institute of Psychiatry, Psychology and Neuroscience, King’s Centre for Military Health Research, King’s College London, London, UK; 3 NIHR Maudsley Biomedical Research Centre, South London and Maudsley Mental Health NHS Trust, London, UK; 4 Department of Biostatistics and Health Informatics, Institute of Psychiatry, Psychology and Neuroscience, King’s College London, London, UK; 5 Centre for Mental Health Policy and Evaluation, Health Service and Population Research Department, Institute of Psychiatry, Psychology, and Neuroscience, King’s College London, London, UK; 6 Department of Occupational Health, Guy’s and St Thomas’ Hospitals NHS Trust, London, UK; 7 Centre for Society and Mental Health, Institute of Psychiatry, Psychology and Neuroscience, King’s College London, London, UK; 8 Academic Department of Military Mental Health, Department of Psychological Medicine, Institute of Psychiatry, Psychology and Neuroscience, King’s College London, London, UK; 9 Department of Sociology, University of Bergen, Bergen, Norway

**Keywords:** Administrative benefits data, Mental disorders, Registry data, Sequence analysis, Social security, Universal credit, Welfare

## Abstract

**Purpose:**

To examine Universal Credit (UC) trajectories, and transitions between UC conditionality regimes among secondary care mental health service users. Sociodemographic and diagnostic characteristics associated with UC trajectories were explored.

**Methods:**

Mental health record data from 4876 individuals who attended mental health services were linked with administrative benefits data. An entry cohort was created including mental health service users who had received UC in 2016 and followed up for 4.5 years. Sequence analysis was used, followed by cluster analysis to identify typical UC trajectories. Sociodemographic and diagnostic characteristics associated with UC clusters were explored using multinominal logistic regression; results were presented as average marginal effects.

**Results:**

Six distinct UC clusters were identified. These clusters indicated: short-term UC searching for work (18.7%), medium-term UC searching for work (19.1%), long-term UC searching for work (21.4%), no work requirements (11.9%), UC working cluster (6.1%), and no work searching and caring responsibilities cluster (22.8%). Women were overrepresented in the medium-term UC searching for work cluster whereas older people were more likely to be in the long-term UC searching for work and no work requirements clusters. Those diagnosed with severe mental illness were overrepresented in the no work requirements group.

**Conclusion:**

Most trajectories were dominated by those required to search for work albeit for different time periods before exiting UC. One in ten people were assessed as unable to work for an extended period. Findings can be used to inform support for people with mental health problems vulnerable to conditionality or longer-term UC receipt.

**Supplementary Information:**

The online version contains supplementary material available at 10.1007/s00127-025-02930-3.

## Introduction

The UK has seen an increase in ill-health and economic inactivity, the latter mainly driven by long-term sickness attributed to mental health problems [1]. This has put pressure on the public purse, in the form of lost tax revenue, labour force shortages, increased welfare expenditure and increased demand for physical and mental health services [2]. Hence the UK has implemented various welfare reforms over the last decade to tackle these challenges [3].

The introduction of Universal Credit (UC) was one such major welfare reform aimed at streamlining and simplifying the welfare system. Additionally, it was hoped it would encourage more people in receipt of benefits to gain and sustain paid employment. The introduction of UC is part of a broad international trend toward transforming welfare systems into what are often labeled as"one-stop shops". These single-entry point services have been introduced to address the fragmentation and coordination problems inherent in welfare services in a range of countries internationally [4].

UC replaced six benefits and tax credits (these are also known as legacy benefits). The implementation of UC started in April 2013, initially in a small number of locations in the North-West of England, followed by a stepwise national roll-out that is expected to be completed in 2028/29 [5, 6].

People who are in work or out of work can claim UC. UC is made up of a standard allowance to reflect the general needs of the claimant and an additional five elements (e.g. childcare element, carer element, housing element) that people could be entitled to depending on their individual or household circumstances [7]. UC is means-tested, i.e. certain financial conditions need to be met as eligibility for UC depends on the income and capital (e.g. savings, investments) of both the recipient and, where relevant, their partner or other household members. If these earnings are above a particular threshold, they are no longer eligible for UC. People also need to accept a ‘claimant commitment’ to be eligible for UC thereby accepting that they are subject to certain requirements and expectations as part of their UC entitlement [8]. These requirements and expectations depend on which of six conditionality regimes someone is allocated to as well as a person’s individual circumstances [9].

The six UC conditionality regimes [9] are listed in Table 1. Individuals in the “searching for work” regime are most likely not in work, or only earn a limited amount. Those in the “working—with requirements” regime are in work, but could earn more, or are not in work but have a partner who does not earn above the earnings threshold. For these two conditionality regimes, individuals are expected to intensively search for work (e.g. up to 35 hours a week), work more hours or find a better paid role.

If a person is allocated to the “no work requirements” regime, they may have health problems or caring responsibilities that prevent them from working, and therefore they are currently not expected to work. UC recipients in the “working—no requirements” group do not earn enough as an individual or as a household for their claim to be withdrawn but earn sufficiently that conditionality does not apply. Individuals in this group may, for example, receive some financial support for childcare or housing costs. For both these groups, no activities or tasks are stipulated that they are required to undertake to continue to receive UC. Individuals in the “planning for work” and “preparing for work” regimes are expected to work in the future but, in general, are prevented by this due to lead parental or caring responsibilities for young children. Depending on which of the two regimes they are in, they may have to attend interviews with a work coach and/or take steps to prepare for work, for example, CV writing or undertaking training.

Little is known about UC conditionality regime trajectories as access to administrative benefits data is highly restricted in the UK. Whereas aggregate benefits data are available in the public domain, individual level data at benefit spell level needed for these kind of analyses are not publicly available [10]. It is likely that UC trajectories differ depending on a variety of personal characteristics such as sex, age, ethnicity and health. For example, previous research has indicated that mental health service users from an ethnic minority background and women were more likely receive UC compared to those from a White background and men respectively [11]. We also know that many mental health service users are allocated to UC regimes that involve conditionality such as the “searching for work conditionality regime” [11].

In contrast to the UK, in Nordic countries access to individual level data across administrative, health and non-health datasets has long been the norm as well as enhancing these datasets with help of data linkage [12, 13]. Research from the Nordic context has demonstrated the value of such data and sequence methods by showing how welfare state services are deeply interconnected, with individual trajectories across systems revealing dynamics that cannot be captured by studying single events or benefit transitions in isolation. These studies underscore how changes in one part of the system—such as tightening access to a particular benefit—can produce ripple effects across others, and how the cumulative experience of navigating these systems reflects broader patterns in welfare state functioning [14]. Our study brings this perspective to the UK for the first time, using newly available register data to explore trajectories through the UC system and beyond.

Therefore, this paper explored the most typical UC trajectories, and transitions between UC conditionality regimes, among people who accessed mental health services at a large mental health service provider in the UK. Sociodemographic and diagnostic characteristics associated with UC trajectories were also determined.

## Methods

### Study population

Individuals were included if they had accessed secondary mental health services from the South London and Maudsley NHS Foundation (SLaM) Trust. SLaM covers an ethnically diverse, urban population of approximately 1.3 million residents across four boroughs in South London. It provides local mental health services to catchment area residents but also has a national specialist mental health service provision.

We selected an entry cohort of those who successfully claimed UC for the first time in 2016 as UC was only fully rolled-out across the four boroughs that SLaM covers from 2015 onwards [15]. Additionally, this ensured a sufficient sample size for the proposed analysis and reasonable duration of follow-up. Only individuals who were aged 16 years or older at the start of 2016, and below state pension age met the study inclusion criteria, as UC is only available to individuals who fall within this age bracket (16–66 years).

### Data source

Deidentified clinical data derived from SLaM electronic health records via the Clinical Record Interactive Search System (CRIS) and successfully linked with administrative benefits data from the Department for Work and Pensions formed the linked data for this study [16]. Data extracted from CRIS included the age and ethnicity of patients and an indicator of deprivation based on patients' home postcodes. Data were extracted as to whether a patient had emergency department presentations in which they were seen by a member of the liaison psychiatry team. This was used as an indicator of mental health symptom severity. Data were extracted about a patient’s first primary psychiatric diagnosis based on the International Classification of Diseases (ICD)—10th revision ‘F codes’ referring to mental and behavioural disorders [17] and diagnosis date. We classified this in two ways: (i) a binary variable indicating whether an individual had any recorded psychiatric diagnosis (vs no recorded diagnosis), and (ii) a binary variable indicating if somebody had a record of severe mental illness (SMI) or no record of these. Individuals may not have had a recorded psychiatric diagnosis for various reasons including a recent presentation to SLaM and as such a diagnosis was not determined yet, individuals disengaged with the service or were determined not to meet the criteria for a psychiatric diagnosis upon assessment. SMI was identified as ICD codes: F2* (schizophrenia-spectrum disorder), F30*/F31* (bipolar affective disorder) and F3* (affective disorder) [18]. Those with no psychiatric diagnosis were grouped in the no SMI group. We created a derived variable that indicated whether patients received their primary psychiatric diagnosis before or after they first received UC.

Details about start and end dates of UC receipt, including conditionality regime allocation, and data on legacy benefit receipt were extracted from the administrative benefits data provided by DWP as well as information on sex and date of death (where relevant).

We used clinical data covering the period 1st January 2007 to 30th June 2020. Although the clinical data window preceded the roll-out of UC nationally and within the SLaM catchment area, we extracted clinical data closest to a patient’s first UC spell to obtain the most relevant data. We extracted legacy benefit receipt information from the administrative data covering 1st January 2007 to 30th June 2020.

### Statistical analyses

The statistical analysis protocol was pre-registered on the Open Science Framework (https://osf.io/h5fzj) and deviations of the protocol are outlined accordingly. For the current paper, findings are only presented for one entry cohort of patients who had accessed SLaM, namely those who successfully claimed UC for the first time in 2016.

Descriptive statistics were used to present a profile of the sociodemographic characteristics of included patients and their primary psychiatric diagnosis status. We used sequence analysis and cluster analysis to identify typical UC trajectories. Sequence analysis is a method used to examine the order and timing of events, enabling the identification and categorisation of complex transitions within longitudinal data. The transition typologies were discerned through a procedure whereby pairwise distances between sequences were calculated followed by a clustering procedure where typical UC trajectories were identified. The distances between the different sequences were calculated using the longest common subsequence (LCS) as we were predominantly interested in the order of the statuses patients experienced and the shared commonalties between the identified sequences [19]. This is in contrast to what we stated in the protocol, as we indicated we would use Optimal Matching procedure but considering this paper’s aims LCS was deemed a better fit. For the clustering procedure, we applied Wards hierarchical clustering combined with Average Silhouette Width (ASW) cluster validation [20]. We also calculated a turbulence indicator for each individual. Turbulence is a composite index capturing distinct state changes, diversity in states, duration over states, number of transitions, and variation in the timing of these (e.g. [21, 22]). Turbulence can, therefore, be used as an indicator of trajectory stability or complexity [23].

The decision on the clustering solution and quality was based on a combination of the average Silhouette coefficient in combination with the ‘face validity’ and interpretability of the clusters. After we identified the optimal cluster solution, univariable and multivariable multinominal logistic regressions were conducted to explore the associations between sociodemographic and diagnostic characteristics and the six clusters identified. Two sets of multivariable regression models were run. In our models to understand factors associated with UC clusters, all sociodemographic and diagnostic characteristics were included. In this model, we included the binary variable indicating whether somebody had a record of any psychiatric diagnosis or not. To understand to what extent SMI is associated with UC clusters, our second model instead included our binary SMI variable. In deviation of the statistical analysis protocol, we opted to present the results from the regression analyses as average marginal effects to aid comparisons, instead of (adjusted) odds ratios.

Data cleaning, manipulation and the multinomial logistic regression were conducted in Stata MP (version 18). R studio and TraMineR was used to conduct sequence analysis and identify the clusters [24].

#### Status alphabet

We identified monthly statuses for a total duration of 49 months, based on the start of a patient’s first spell in 2016. We identified 9 monthly statuses (Table 1). These were informed by the six conditionality regimes patients could be allocated to and UC eligibility criteria. The amount of UC a recipient receives can fluctuate monthly as it is based on a person’s earnings. Furthermore, if a person experiences changes in their circumstances, the conditionality regime may change accordingly. The UC data available for analysis are taken monthly, namely as registered in the system on the second Thursday of each month. These monthly time points formed the basis of the sequence analysis. If two or more statuses were overlapping in the same month, the first status was prioritised. This was only the case for a few participants. Once a patient reached State Pension age or died, this status would be carried forward over the remaining duration of follow-up.

**Table 1:** Status alphabet

Status	Description^a^
UC—searching for work	Registered as in receipt of UC—searching for work current month
UC—working with requirements	Registered as in receipt of UC—working with requirements current month
UC—no work requirements	Registered as in receipt of UC—no work requirements current month
UC—working no requirements	Registered as in receipt of UC—working no requirements current month
UC—preparing for work	Registered as in receipt of UC—preparing for work current month
UC—planning for work	Registered as in receipt of UC—planning for work current month
Reached State Pension age	Reached State Pension age (66 years) in current month
Death	Registered as died in current month
Other	No evidence of above listed statuses present

^a^Please refer back to the introduction for a detailed description of the UC conditionality regimes

## Results

The sample consisted of 4876 patients. The mean age at entry was 34.04 (SD 11.71), and there were slightly more males (53.5%) than females. Most patients were of a White ethnic background (46.9%) followed by those of a Black/African/Caribbean/Black British ethnic background (19.3%). 70.5% of patients were in the two most deprived Index of Multiple Deprivation (IMD) quintiles. A large proportion of the sample (87.5%) had received legacy benefits before receiving UC (Table 2).

**Table 2:** Sample profile and sociodemographic and diagnostic characteristics of the six typical UC clusters (n = 4876)

Characteristics	Total, n (%)	Cluster 1: Short-term searching for work, n (%)	Cluster 2: Medium-term searching for work, n (%)	Cluster 3: Long-term searching for work, n (%)	Cluster 4: No work requirements, n (%)	Cluster 5: Working cluster, n (%)	Cluster 6: No work searching and caring responsibilities, n (%)
Overall	4876 (100.0)	913 (18.7)	931 (19.1)	1045 (21.4)	579 (11.9)	296 (6.1)	1112 (22.8)
Sex							
Female	2267 (46.5)	376 (41.2)	341 (36.6)	362 (34.6)	328 (56.6)	210 (71.0)	650 (58.5)
Male	2609 (53.5)	537 (58.8)	590 (63.4)	683 (65.4)	251 (43.4)	86 (29.0)	462 (41.6)
Age (years)^a^							
Mean (SD)	34.04 (11.71)	30.30 (10.73)	32.35 (11.33)	35.45 (11.50)	37.62 (12.28)	36.54 (9.26)	34.69 (12.24)
16–24	1249 (25.6)	345 (37.8)	290 (31.2)	217 (20.8)	97 (16.8)	25 (8.5)	275 (24.7)
25–34	1530 (31.3)	309 (33.8)	299 (32.1)	313 (30.0)	164 (28.3)	101 (34.1)	344 (30.9)
35–44	968 (19.9)	125 (13.7)	167 (17.9)	230 (22.0)	124 (21.4)	115 (38.9)	207 (18.6)
45–54	855 (17.5)	109 (11.9)	135 (14.5)	235 (22.5)	130 (22.5)	46 (15.5)	200 (18.0)
55–66	274 (5.6)	25 (2.7)	40 (4.3)	50 (4.8)	64 (11.1)	< 10 (3.0)	86 (7.7)
Ethnicity							
White	2285 (46.9)	439 (48.1)	416 (44.7)	459 (43.9)	291 (50.3)	147 (49.7)	533 (47.9)
Black/African/Caribbean/Black British	942 (19.3)	141 (15.4)	206 (22.1)	254 (24.3)	94 (16.2)	51 (17.2)	197 (17.7)
Asian/Asian British	266 (5.5)	47 (5.2)	47 (5.1)	57 (5.5)	33 (5.7)	23 (7.8)	59 (5.3)
Mixed/Multiple racial and ethnic groups	169 (3.5)	27 (3.0)	30 (3.2)	34 (3.3)	26 (4.5)	< 10 (3.0)	43 (3.9)
Other racial and ethnic minority groups	357 (7.3)	66 (7.2)	52 (5.6)	72 (6.9)	50 (8.7)	23 (7.8)	94 (8.5)
Not stated	856 (17.6)	193 (21.1)	180 (19.3)	169 (16.2)	85 (14.7)	43 (14.5)	186 (16.7)
Deprivation (IMD quintile)^b^							
First (most deprived)	1659 (34.0)	263 (28.8)	310 (33.3)	385 (36.8)	220 (38.0)	94 (31.8)	387 (34.8)
Second	1778 (36.5)	314 (34.4)	343 (36.8)	369 (35.3)	199 (34.4)	117 (39.5)	436 (39.2)
Third	731 (15.0)	169 (18.5)	138 (14.8)	147 (14.1)	80 (13.8)	49 (16.6)	148 (13.3)
Fourth	307 (6.3)	72 (7.9)	62 (6.7)	54 (5.2)	35 (6.0)	19 (6.4)	65 (5.9)
Fifth (least deprived)	166 (3.4)	52 (5.7)	39 (4.2)	22 (2.1)	16 (2.8)	< 10 (3.0)	28 (2.5)
Not stated	235 (4.8)	43 (4.7)	39 (4.2)	68 (6.5)	29 (5.0)	< 10 (2.7)	48 (4.3)
Primary psychiatric diagnosis recorded (ICD-10 codes)^c^							
No	1476 (30.3)	316 (34.6)	311 (33.4)	329 (31.5)	128 (22.1)	96 (32.4)	296 (26.6)
Yes	3400 (69.7)	597 (65.4)	620 (66.6)	716 (68.5)	451 (77.9)	200 (67.6)	816 (73.4)
Severe mental illness (SMI) diagnosis (F2* (schizophrenia-spectrum disorder), F30*/F31* (bipolar affective disorder) and F3* (affective disorder))							
No SMI diagnosis recorded	4098 (84.0)	799 (87.5)	789 (84.8)	911 (87.2)	425 (73.4)	254 (85.8)	920 (82.7)
SMI diagnosis recorded	778 (16.0)	114 (12.5)	142 (15.2)	124 (12.8)	154 (26.6)	42 (14.2)	192 (17.3)
Liaison psychiatry referral during emergency department presentation							
No	3798 (77.9)	729 (79.9)	731 (78.5)	828 (79.2)	412 (71.2)	253 (85.5)	845 (76.0)
Yes	1078 (22.1)	184 (20.1)	200 (21.5)	217 (20.8)	167 (28.8)	43 (14.5)	267 (24.0)
If yes, number of referrals (median (IQR))	1 (1,1)	1 (1,1)	1 (1,2)	1 (1,2)	1 (1,3)	1 (1,1)	1 (1,2)
Received UC before/after primary psychiatric diagnosis^d^							
UC before primary psychiatric diagnosis	1403 (28.8)	225 (24.6)	254 (27.3)	276 (26.4)	225 (38.9)	45 (15.5)	377 (33.9)
UC received after primary psychiatric diagnosis	1996 (40.9)	372 (40.7)	366 (39.3)	439 (42.0)	226 (39.0)	154 (52.0)	439 (39.5)
No psychiatric diagnosis received	1476 (30.3)	316 (34.6)	311 (33.4)	329 (31.5)	128 (22.1)	96 (32.4)	296 (26.6)

*UC* Universal Credit, *IMD* Index of Multiple Deprivation

^a^Calculated/determined at first UC spell start date

^b^Informed by postcode active on or closest before or after a patient’s start date of their first UC spell using 2015 IMD data

^c^Closest to the start date of first UC spell. & administrative benefits data coverage window ranges from 1st January 2005 till 30th June 2020 (including Housing Benefit, Employment and Support Allowance, Job Seeker’s Allowance and Income Support)

^d^1 person had the same date for first UC

### Description of typical UC trajectory clusters and associated characteristics

**Fig. 1:**
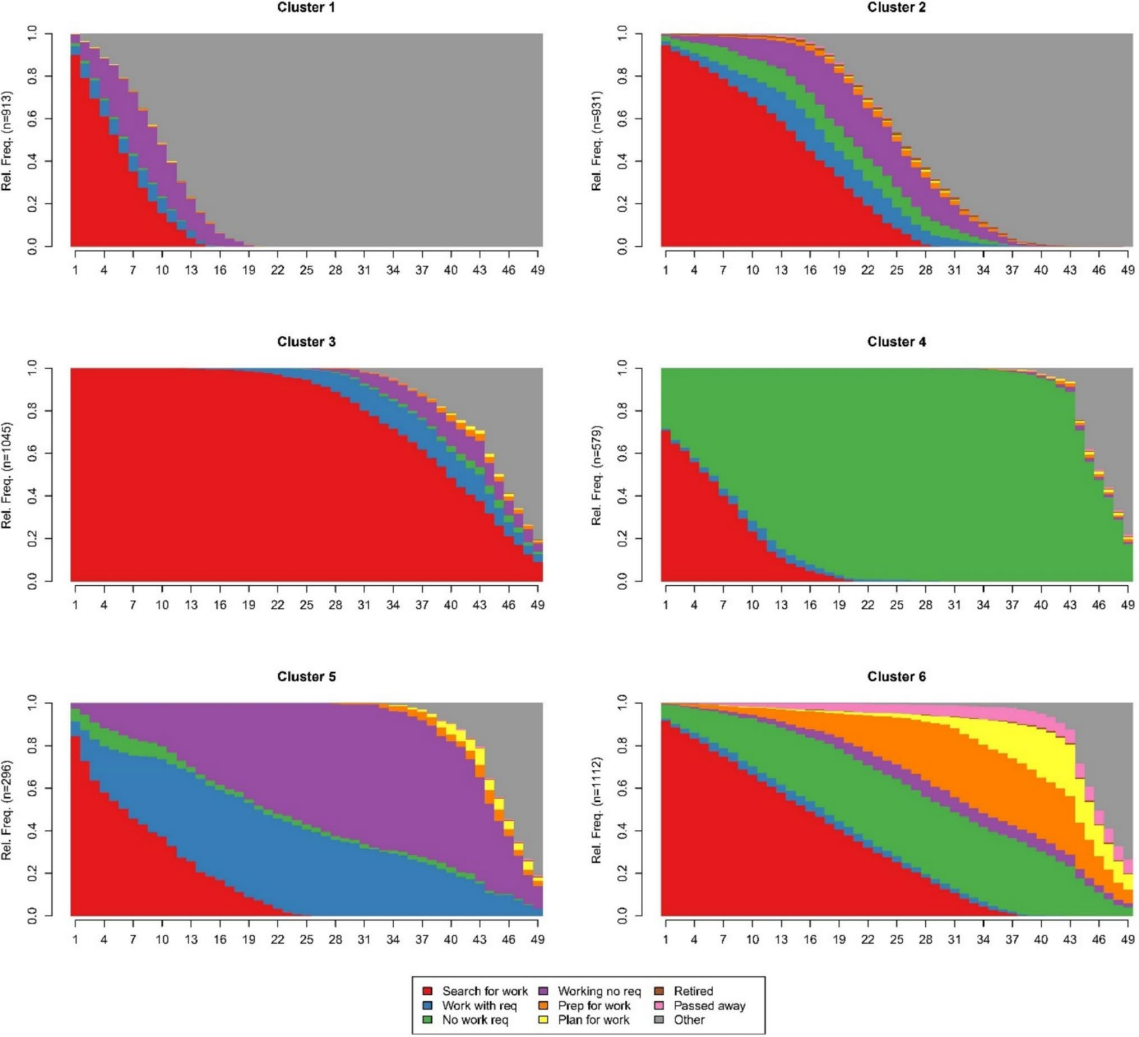
Chronogram of 6 distinct typical UC clusters

**Fig. 2:**
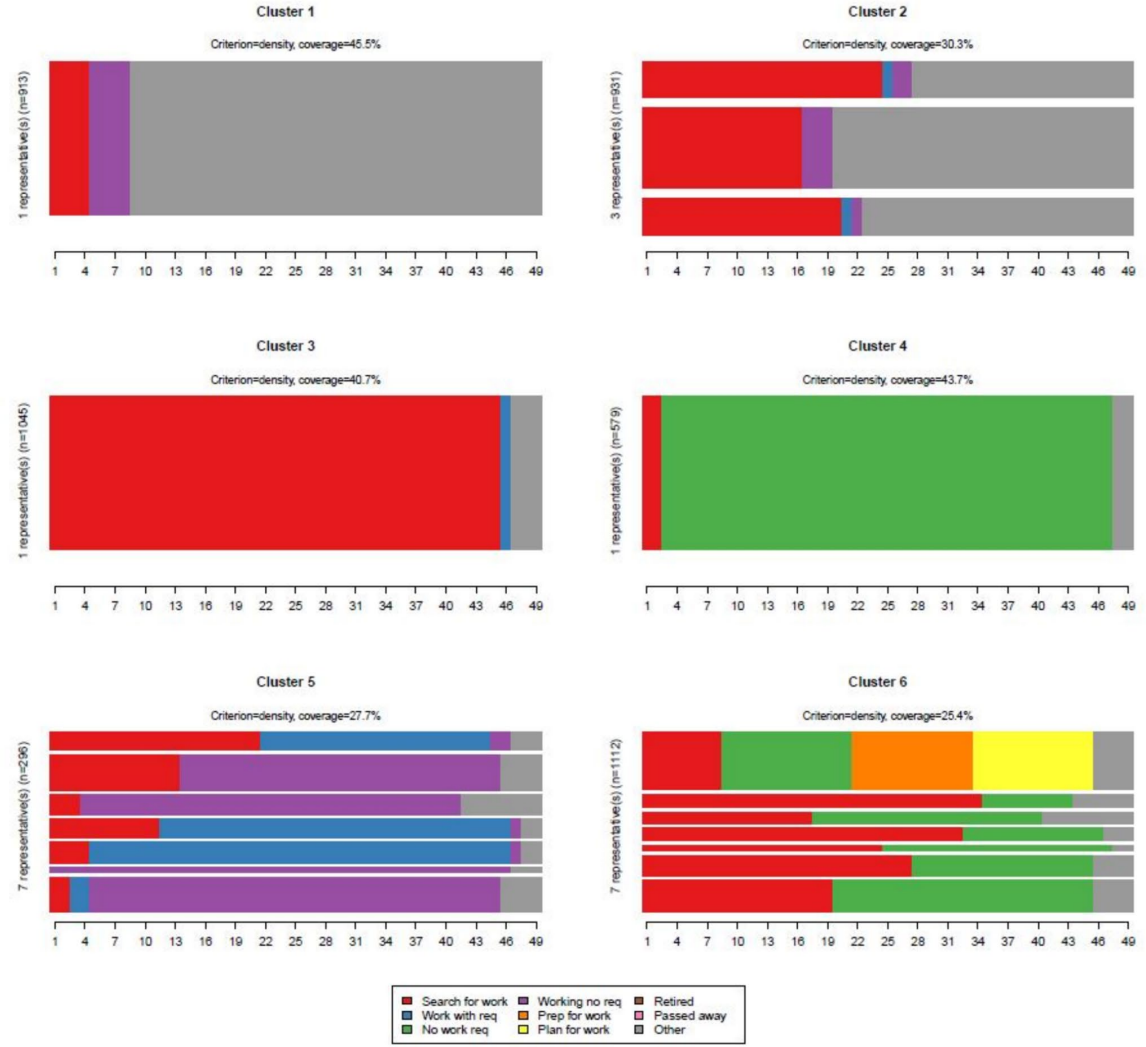
Representative sequence plots for typical UC clusters

The optimal matching and clustering procedure identified six unique UC trajectory types. Figure 1 shows the chronogram of each cluster, depicting the aggregated relative frequency of each UC regime state over time. Representative sequence plots can be found in Fig. 2. An overview of the sociodemographic and diagnostic characteristics for the six clusters are shown in Table 2 and a summary description of the different clusters in Table 3. Mean time in each state and total time in UC states by cluster are presented in Supplementary Fig. 1. Effect plots from the multinominal logistic regression are presented in Supplementary Fig. 2–3 as average marginal effects (AME).

**Table 3:** Summary of typical UC clusters

Cluster	Description of trajectory	Key sociodemographic and characteristics of cluster	% (n)
Cluster 1: Short-term UC searching for work	In the UC searching for work or UC working no requirements for up to 12–18 months and then exit UC *Mean time in receipt of UC: 9.23 months (SD* = *4.21)* *Mean time in UC searching for work: 5.20 months (SD* = *3.78)*	Predominantly younger adults Higher proportion from lesser deprived areas Black/Black British ethnic groups less likely to be in this group	18.7 (913)
Cluster 2: Medium-term UC searching for work	A complex cluster with various trajectories whereby most people remain in the UC searching for work and precarious work regimes for over 18 months, but all exit UC within 3 years *Mean time in receipt of UC: 24.99 months (SD* = *6.6X)* *Mean time in UC searching for work: 13.86 months (SD* = *7.74)*	More men than women Younger age groups more represented Black/Black British ethnic groups more likely to be in this group	19.1 (931)
Cluster 3: Long-term UC searching for work	In UC searching for work regime for more than 2 years with little representation of the UC no work requirement regime *Mean time in receipt of UC: 43.54 months (SD* = *4.98)* *Mean time in UC searching for work: 38.14 months (SD* = *8.05)*	People aged 35–55 more represented More men than women From areas with higher deprivation Black/Black British ethnic groups more likely to be in this group	21.4 (1045)
Cluster 4: No work requirements	The majority of those in this cluster remain in the UC no work requirements cluster indicating they are likely unable to work due to health reasons *Mean time in receipt of UC: 45.62 months (SD* = *2.75)* *Mean time in UC searching for work: 5.51 months (SD* = *5.25)* *Mean time in UC no work requirements: 39.02 months (SD* = *6.39)*	SMI diagnosis highly represented in this cluster Increasing likelihood to be in this cluster with increasing age	11.9 (579)
Cluster 5: Working cluster	Most individuals in this cluster are in the working with requirements or working no requirements regime (i.e. they are employed but receiving some element of UC) *Mean time in receipt of UC: 44.60 months (SD* = *3.63)* *Mean time in UC searching for work: 7.42 months (SD* = *6.85)* *Mean time in UC working with requirements: 14.30 months (SD* = *14.03)* *Mean time in UC working no requirements: 20.21 months (SD* = *14.39)*	Women make up 71% of this group Mostly aged 25–44 years	6.1 (296)
Cluster 6: No work searching and having caring responsibilities	A complex cluster featuring a variety of UC regimes designed for those not currently working or not searching for work. A high proportion of planning and preparing for work states are seen in this group indicating those with caring responsibilities are highly represented *Mean time in receipt of UC: 43.11 months (SD* = *8.13)* *Mean time in UC searching for work: 15.67 months (SD* = *10.62)* *Mean time in UC no work requirements: 12.76 months (SD* = *9.49)* *Mean time in UC preparing for work: 8.09 months (SD* = *10.46)* *Mean time in UC planning for work: 3.25 months (SD* = *5.39)*	More women than men Slightly more likely to have a psychiatric diagnosis than other clusters	22.8 (1112)

*UC* Universal Credit, *SD* standard distribution

**Cluster 1** is the “*short-term UC searching for work”* cluster. It represents 18.7% of the sample (n = 913). It groups people who primarily are in the searching for work, and working no requirements regime, and who transition out of UC receipt within 12–18 months. By 21 months, 100% of people in this cluster are no longer receiving UC.

Cluster 1 is predominantly made up of younger adults, with 71.6% of this group aged under 35 years. The AME model shows that older age groups are significantly less likely to be in this cluster than the youngest age group (Supplementary Fig. 2). This cluster has the highest proportion of people living in the two least deprived areas (13.6%). Black people were 3.9 percentage points less likely to be represented within this cluster than those of a White ethnicity. This cluster had the lowest proportion of people with a psychiatric diagnosis recorded (65.4%) across all clusters, and the highest proportion of people across all clusters who did not receive legacy benefits (21.7%).

**Cluster 2** features varied and complex trajectories, with people in this cluster generally leaving the UC system more quickly than other clusters (except Cluster 1). It represents 19.1% of the sample (n = 931). This cluster describes people who spent some time in the searching for work regime before moving to another UC regime for some time before exiting UC. By month 30 (2.5 years after first receiving UC), 72.7% of this group are no longer receiving UC. This cluster could be described as “*medium-term UC searching for work”.*

There were fewer distinctive differences across characteristics observed within this cluster. As with most other clusters, males are more likely than females to be represented in this cluster. Cluster 2 has a relatively low age profile, meaning that younger people (< 34 years) are more likely to be found in this cluster than older people (Supplementary Fig. 2). Those of Black/African/Caribbean/Black British ethnicity have a 3.9% higher probability of being represented in this cluster than those of White ethnicity.

**Cluster 3** is the “*long-term UC searching for work”* cluster representing 21.4% of the sample (n = 1045). Everyone in this cluster spends approximately the first year in the searching for work regime. At 24 months, 95.3% of this group are still in the searching for work regime, and gradually move to other work-related regimes (working with requirements; working no requirements) or exit UC for the remainder of the observation period. At month 49, 18.2% are still in receipt of UC (9% of which are in the searching for work regime).

In this cluster, people aged 35–44 and 45–54 years were significantly more likely to be represented than younger age groups. 72.1% of people in this cluster were living in the two most deprived areas. The AME models corroborated a statistically significant difference by deprivation. As in Cluster 1, the only significant difference observed for ethnicity is seen in the Black/African/Caribbean/Black British ethnicity group. Cluster 3 has the highest proportion of people who have claimed legacy benefits (93.8%) across all clusters.

**Cluster 4** is the “*no work requirements”* cluster representing 11.9% of the sample (n = 579). This cluster describes people who spend long periods of time (M: 39 months) in the no work requirements UC regime.

Cluster 4 has an older demographic, with the highest percentage of people in the oldest age group (11.1%) across all clusters. The AME shows a gradient whereby the percentage point increase of representation in the cluster increases with increasing age. This cluster has the highest percentage of those living in the most deprived quintile (38.0%) compared to other clusters as well as having a primary psychiatric diagnosis (77.9%), and emergency department liaison psychiatry presentations (28.8%). Females have a 5.4% higher probability than men to be represented in this cluster.

**Cluster 5**, while representing varied individual sequences, could be referred to as the “*working cluster”*. It represents 6.1% of the sample (n = 296). This cluster describes people who mostly spend notable periods of time in working states, namely the UC working with requirements or the working no requirements regime.

This cluster has the highest percentage of females (71.0%) and 73.0% of people are aged 25–44 years. It has the lowest percentage of people who have presented to the emergency department (14.5%) than any other cluster. There were no observed statistical differences in ethnicity or deprivation.

**Cluster 6** is a complex group, featuring a variety of UC regimes designed for those not currently working or searching for work (“*no work searching and having caring responsibilities”* cluster) representing 22.8% of the sample (n = 1112). This cluster has the most substantial representation of planning for work and preparing for work regimes across all the UC clusters, with over 50% of patients falling within these two regimes at times within the observation period.

Cluster 6 has more females than males, with females 11.1 percentage points more likely than men to be represented in this cluster. 73.4% of people represented in this cluster have a primary psychiatric diagnosis. There were no observed differences by ethnicity or deprivation within this cluster. A statistical difference was reported in the age profile of this cluster, with people in the oldest age group having a 10.1% higher probability of being in Cluster 6 than those in the youngest age group.

#### Severe mental illness diagnosis and typical UC clusters

The results of our analysis exploring how an SMI diagnosis is related to UC clusters are shown in Supplementary Fig. 3, and descriptive statistics are reported in Table 2. We found Cluster 4 (*“no work requirements”)* had a particularly high concentration of people with an SMI diagnosis (26.6%). The AME model indicated that people with an SMI diagnosis had an 8.9% higher probability of being represented in this cluster than those with no SMI diagnosis. This analysis found that those in Cluster 1 (*“short-term UC searching for work”)* and Cluster 3 (*“long-term UC searching for work”)* were significantly less likely to have an SMI diagnosis. The AME model showed that people with an SMI diagnosis had a 3.1% lower probability of being represented in Cluster 1 than those without an SMI diagnosis. People with an SMI diagnosis had a 5.8% lower probability of being represented in Cluster 3 than those without an SMI diagnosis.

## Discussion

In a cohort of people who accessed secondary mental health services and who received UC for the first time in 2016, we found six typical UC trajectory clusters over a follow-up period of approximately 4.5 years. Most clusters were dominated by UC regimes that are subjected to conditionality, particularly involving requirements to search for work to maintain their entitlement to receive financial support via UC. These clusters were distinct from each other by the approximate maximum amount of time an individual spent in the ‘searching for work’ UC regime ranging from, for example, short-term (up to 14 months—Cluster 1), medium-term (up to 29 months—Cluster 2), long-term (up to 49 months—Cluster 3) before flowing off UC. These three searching for work clusters covered 59.2% of the sample.

Some distinct differences were found in the profile of individuals who followed these typical trajectories whereby the “*short-term UC searching for work”* cluster was dominated by younger, White individuals and those living in less deprived areas who were less likely to have received legacy benefits. This contrasted with the *“medium and long-term UC searching for work”* clusters that included individuals living in more deprived areas and it was observed that those from a Black ethnic background were overrepresented in both clusters, affirming previous research indicating additional labour market barriers for this group of individuals [25, 26]. Older people were more commonly identified in the “*long-term UC searching for work*” cluster. We know that the number of work-limiting conditions increases with age [27]. Despite this, many workers in their 50 s and 60 s are working and other barriers such as ageism, stigma and caring responsibilities are likely to hinder work participation [28, 29]. As such, it is imperative that employers and government continue to work together to break down barriers for this important, and growing, group of workers.

We also know that the longer someone is out of work, the less likely they are to return to the workplace [30]. Therefore, a particular focus should be directed at people in the *“medium and long-term UC searching for work*” clusters to ensure that they do not lose their connection with the labour market; upstream prevention methods and early intervention initiatives aimed at these individuals should be a priority to prevent them from falling out of work in the first place [31]. This could include flexibility around working hours, workplace adjustments and support with managing (mental) health and wellbeing in the workplace [31]. However, the onus should not be placed solely on the individual. It is important, and beneficial from a business perspective, that employers facilitate a supportive, holistic, inclusive workplace facilitating the mental health needs of their workers [32].

We found that individuals in “*long-term UC searching for work”* were no more likely to be diagnosed with an SMI or other psychiatric diagnosis compared to those in other clusters, indicating that their mental health may not be the primary reason for their worklessness, but other factors may be at play (e.g. local labour market opportunities, affordable childcare options, skills gap). We found one UC cluster (Cluster 4) in which individuals were, in general, not subjected to conditionality and this covered 11.9% of the sample. This *“no work requirements”* cluster includes those assessed as unable to work (or with caring responsibilities for a child aged under 1). In general, people in this cluster were allocated to the “no work requirements” UC regime for an extended duration of time (> 36 months) after spending a brief period searching for work. The latter is likely because people must undergo a work capability assessment (WCA), and await the decision, before they get allocated to the “no work requirements” UC regime. The WCA has been criticised for being mainly focused and tailored to long-standing physical health conditions, and less suitable to the work-limiting impacts of fluctuating, chronic mental health conditions [33]. Nevertheless, we found that people with an SMI or other psychiatric diagnoses were overrepresented in this cluster.

In the smallest UC cluster (6.1%; Cluster 5—*“working cluster”*) middle-aged females (aged 25–44 years) living in the two most deprived areas were overrepresented and they were often allocated to the “working no requirements” or “working with requirements” UC regime over the course of follow-up. In general, this means that they were in work, but most likely lower paid or part-time work. Therefore, they were earning under a certain threshold and remained eligible to receive financial support via UC. On-going initiatives by the UK Government including the latest commitment to increase the national minimum wage from April 2025 as well as an expansion of financial support to cover childcare costs might be particularly relevant for this group [34]. This could possibly allow them to work more hours or find a better paid job, and subsequently increase their income and exit UC.

The last UC cluster we identified (i.e*.* Cluster 6—*“no working searching and caring responsibilities”*) was the most sizeable cluster (22.8%) and varied in its make-up. This cluster consisted mainly of older females who had been expected to search for work for some time, before taking up caring responsibilities meaning they were no longer required to search for work or had been assessed as unfit for work but only for a limited period (max. 12–15 months). This group would benefit from targeted initiatives that focus on those who are out of work, whether due to ill health or caring responsibilities, that enables them to maintain the connection with the workplace to facilitate a smooth transition back when they can.

A major goal of the UK Government when designing the UC system was to support people into employment, with the intention of ensuring people are better off financially when in work than remaining on benefits (‘making work pay’ [35]). We found that across all UC clusters, a large proportion of the sample were no longer in receipt of UC in the final month of observation (month 49). This ranged from 73.4% (Cluster 6) to 100% (Cluster 1 and Cluster 2).

### Strengths and limitations

The strength of this study lies in the type, size and nature of the data used, namely high-quality individual level administrative benefits data linked with mental health record data. Furthermore, the benefits data window covered a period of welfare reform during which UC was implemented nationally [36]. The application of sequence analysis allowed for a holistic exploration of multiple, complex transitions over time that are likely to interact instead of only focusing on single transitions, thereby making optimal use of the longitudinal, dynamic nature of the benefits data. Our diagnostic data were limited by their temporality, as we only modelled a patient’s first psychiatric diagnosis. Occasionally this meant that the duration between first UC receipt, SLaM referral and diagnosis was a distinct period apart. Nevertheless, mental disorders, especially those at the more severe end of the spectrum as seen in a secondary mental health service like SLaM, are often chronic in duration and may have had a negative impact on a person’s functioning for a longer period, whether in the lead up to a mental health service referral, or after having received a diagnosis. Additionally, we lacked information on whether individuals indeed found employment after they stopped receiving UC or whether there were other reasons such as disengagement with the welfare system or changes in household composition that resulted in UC off flow.

## Conclusions

We identified six typical UC trajectory clusters among individuals who accessed secondary mental health services and were first time UC claimants. Most trajectories were dominated by a UC regime that required individuals to search for work, albeit for various durations, before flowing off UC. The sociodemographic and diagnostic profile of individuals changed across these work-search focused UC trajectories indicating different distances to the labour market that likely interacted with personal circumstances. Only one in ten mental health service users were allocated to a trajectory that indicated they had been assessed as unable to work most likely due to ill health.

Our findings provide valuable insights regarding the characteristics of UC claimant groups who have accessed mental health services and are at an increased risk of falling out of work for a longer period. This evidence can be used to tailor interventions to break down barriers that hinder a timely return to work for those who are able to, as well as tailoring initiatives that prevent people from falling out of work in the first place. It also provides further evidence to inform public and welfare policy to ensure that the needs of individuals affected by mental health problems are duly considered when they engage with the welfare system.

## Supplementary Information

Below is the link to the electronic supplementary material.

Supplementary file1 (DOCX 236 KB)

## Data Availability

Maudsley Biomedical Research Centre at the South London and Maudsley NHS Foundation Trust, upon reasonable request. Requests for data will be considered on a case-by-case basis, given the sensitive nature of the data, and access will only be granted if approval is given by the Work and Health Screening Panel and other governance requirements are fulfilled. For more information, please contact: cris.administrator@slam.nhs.uk.
