# Supplementary material for: Universal credit trajectories among individuals who access secondary mental health services: analysis of linked data
Source: Soc Psychiatry Psychiatr Epidemiol. 2025 May 30;61(1):53–66. doi: 10.1007/s00127-025-02930-3 (PMC12855221; doi:10.1007/s00127-025-02930-3)
Supplement: Supplementary file 1 — Supplementary file1 (DOCX 236 KB) [file 127_2025_2930_MOESM1_ESM.docx]

**Universal Credit trajectories among individuals who access secondary mental health services: analysis of linked data.**

Sharon A.M. Stevelink [1,2]*, Sarah Ledden [1]*, Ioannis Bakolis [3-5], Ray Leal [1,2], Ira Madan [6], Matthew Hotopf [1,3], Nicola T. Fear [2,8], Thomas Lorentzen [9].

1. Department of Psychological Medicine, Institute of Psychiatry, Psychology and Neuroscience, King’s College London, London, UK.
2. King’s Centre for Military Health Research, Department of Psychological Medicine, Institute of Psychiatry, Psychology and Neuroscience, King’s College London, London, UK.
3. NIHR Maudsley Biomedical Research Centre, South London and Maudsley Mental Health NHS Trust, London, UK.
4. Department of Biostatistics and Health Informatics, Institute of Psychiatry, Psychology and Neuroscience, King’s College London, London, UK.
5. Centre for Mental Health Policy and Evaluation, Health Service and Population Research Department, Institute of Psychiatry, Psychology, and Neuroscience, King’s College London.
6. Department of Occupational Health, Guy’s and St Thomas’ Hospitals NHS Trust, London, UK.
7. Centre for Society and Mental Health, Institute of Psychiatry, Psychology and Neuroscience, King’s College London, London, UK.
8. Academic Department of Military Mental Health, Department of Psychological Medicine, Institute of Psychiatry, Psychology and Neuroscience, King’s College London, London, UK.
9. Department of Sociology, University of Bergen, Bergen, Norway.

**Joint first authors*

*Corresponding author:* Dr Sharon Stevelink - [Sharon.stevelink@kcl.ac.uk](mailto:Sharon.stevelink@kcl.ac.uk).

*ORCID:*

Sharon A.M. Stevelink: 0000-0002-7655-7986; Ioannis Bakolis: 0000-0002-4800-1630; Ray Leal: NA; Sarah Ledden: 0000-0002-5121-7943; Ira Madan: 0000-0003-2200-7329; Matthew Hotopf: 0000-0002-3980-4466; Nicola T. Fear: 0000-0002-5792-2925; Thomas Lorentzen: 0000-0001-9952-8928.

**SUPPLEMENTARY MATERIALS**


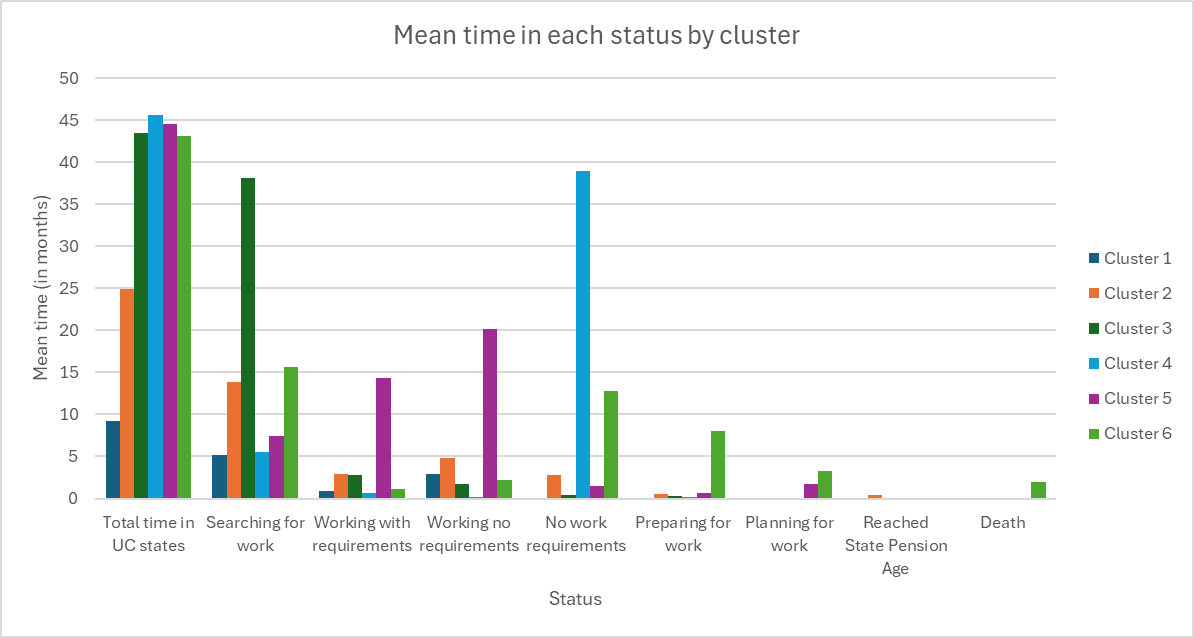
*Supplementary Figure 1: Mean time in each status by cluster*


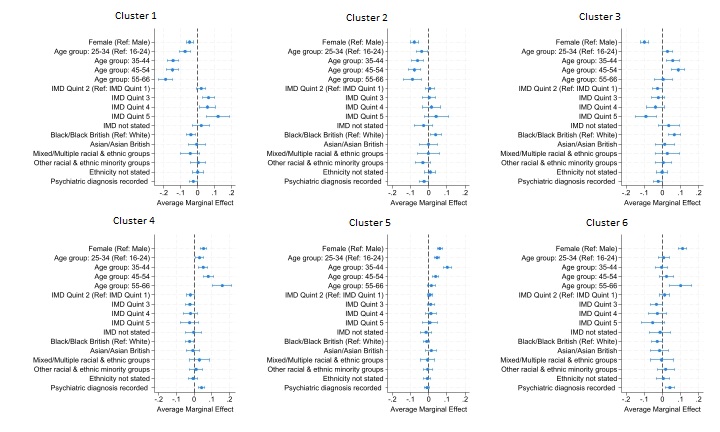


*Supplementary Figure 2: Average Marginal Effects for sociodemographic and diagnostic characteristics for UC clusters* *.*


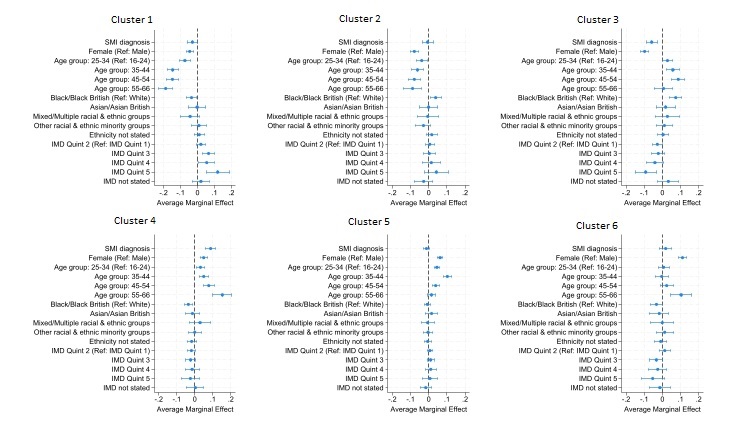


*Supplementary Figure 3: Average Marginal Effects for adjusted analysis testing associations between Severe Mental Illness diagnosis and UC clusters.*
